# Autoantibodies against tumor‐associated antigens combined with microRNAs in detecting esophageal squamous cell carcinoma

**DOI:** 10.1002/cam4.2792

**Published:** 2019-12-19

**Authors:** Guiying Sun, Hua Ye, Xiao Wang, Tiandong Li, Di Jiang, Cuipeng Qiu, Liping Dai, Jianxiang Shi, Kaijuan Wang, Chunhua Song, Peng Wang, Jianying Zhang

**Affiliations:** ^1^ College of Public Health Zhengzhou University Zhengzhou China; ^2^ Henan Key Laboratory of Tumor Epidemiology Zhengzhou University Zhengzhou China; ^3^ Henan Institute of Medical and Pharmaceutical Sciences Zhengzhou University Zhengzhou China; ^4^ Department of immunology College of Basic Medicine Zhengzhou University Zhengzhou China

**Keywords:** autoantibodies, diagnosis, esophageal squamous cell carcinoma, microRNAs

## Abstract

Esophageal carcinoma (EC) is a common malignant disease worldwide, especially in China. There is currently no specific blood test for detecting EC. Autoantibodies against tumor‐associated antigens (TAAbs) and microRNAs (miRNAs) are promising markers for cancer diagnosis and this study focuses on combining TAAbs and miRNAs to evaluate the diagnostic value in esophageal squamous cell carcinoma (ESCC). The expression levels of seven TAAbs and five microRNAs in plasmas from 125 patients diagnosed with ESCC and 125 healthy individuals were detected by enzyme‐linked immunosorbent assay (ELISA) and reverse transcription quantitative‐polymerase chain reaction (RT‐qPCR), respectively. The receiver operating characteristic (ROC) analysis was applied to estimate the diagnostic value of these markers for distinguishing ESCC patients from normal individuals. Logistic regression analysis was performed to generate prediction model and calculate the probability of individuals being diagnosed with ESCC. Three panels were established including four TAAbs, three miRNAs, and three TAAbs combined with three miRNAs. The panel consisting of three TAAbs (HCCR, C‐myc, and MDM2) and three miRNAs (miR‐21, miR‐223, and miR‐375) attained great diagnostic value for ESCC, with an area under the receiver operating characteristic curve (AUC) of 0.89 (95% CI: 0.85‐0.93) with the sensitivity of 69%, the specificity of 90%, the PPV of 83%, the NPV of 79%, and the coincidence rate of 81%. The optimal panel of six‐member markers was able to effectively discriminate the patients with ESCC from normal individuals, especially for early esophageal squamous cell carcinoma.

## Graphical abstract

The panel consisting of three TAAbs (HCCR, C‐myc, and MDM2) and three miRNAs (miR‐21, miR‐223, and miR‐375) attained great diagnostic value for ESCC, with an area under the receiver operating characteristic curve (AUC) of 0.89 (95% CI: 0.85‐0.93) with the sensitivity of 69%, specificity of 90%, and the coincidence rate of 81%. Our results reveal that using the combination of microRNAs and TAAbs may aid to diagnose ESCC, especially for ESCC at the early stage.

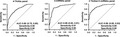

## 1 INTRODUCTION

Esophageal cancer (EC) is one of the most common malignant tumors of the digestive tract, ranking seventh in the incidence and sixth among the death of malignant tumor worldwide.^1^ According to the latest data of the global cancer epidemic, there were 572 000 new cases and 509 000 death cases of esophageal cancer in 2018 worldwide. China was one of the five regions with the highest incidence of esophageal cancer in the world.^2^ In China, the incidence of esophageal cancer ranks sixth in the incidence of malignant tumors, and the mortality rate ranks fourth in the cause of death of malignant tumors according to the latest cancer statistics.^3^ Esophageal cancer mainly includes esophageal squamous cell carcinoma (ESCC) and esophageal adenocarcinoma (EAC). In China, ESCC accounts for more than 90% of esophageal cancer.^4^

Esophageal cancer has a poor prognosis with the 5‐year survival rate of less than 20%.^5^ Studies have shown that the 5‐year survival rate of early patients with EC was up to 80%‐90%,^6^, ^7^ which indicates early diagnosis is essential for improving the survival rate of EC patients. However, early detection is hampered by the lack of typically clinical symptoms and reliable noninvasive screening methods. Autoantibodies against tumor‐associated antigens (TAAbs) are the antigenic protein produced by tumor cells, which can trigger an immune response in patients diagnosed with cancer. The level of anti‐TAAs autoantibodies in plasma of patients has a tendency to rise earlier than the appearance of clinical symptoms.^8^ Many studies have illustrated that autoantibodies may be promising as biomarkers for detecting cancer, for instance, colorectal cancer,^9^ hepatocellular carcinoma,^10^ and lung cancer.^11^ The combination of TAAbs can improve the performance in the diagnosis of cancers.^12^ Looi et al reported that the panel of three TAAbs (p16, C‐myc, and p53) achieved the sensitivity of 22.5% and specificity of 97.6% in discriminating EC from normal individuals.^13^ MicroRNA (miRNA) is a kind of noncoding small RNA containing about 22 nucleotides. In 2005, a study published in Nature reported that miRNAs can accurately classify tumors and are more accurate than the expression profiles of mRNA.^14^ In 2008, Lawrie,^15^ Mitchell,^16^ and Chen^17^ reported the discovery of microRNAs in serum/plasma at almost the same time. Recently, researchers have poured attention into the potential application of miRNAs for detecting cancer.^18^, ^19^, ^20^, ^21^, ^22^ However, the diagnostic value of the combination of single type biomarkers is not enough for ESCC patients, and we try to combine TAAbs with miRNAs to enhance the diagnostic performance of ESCC.

In previous study, our laboratory has tested the level of 15 TAAbs screened by serological proteome analysis (SERPA) and systematic review. A panel of seven TAAbs (p53, p62, HCCR, C‐myc, MDM2, hnRNPA2B1, and NICD), for the detection of ESCC with the highest coincidence rate for detection of ESCC, was established by logistic regression analysis and step‐by‐step optimization. Our previous research showed that the five miRNAs (miRNA‐21, miRNA‐375, miRNA‐223, miRNA‐100, and miRNA‐25) had better diagnostic ability for patients with esophageal cancer.^23^ In the present study, we evaluated the diagnostic value of seven TAAbs (p53, p62, HCCR, C‐myc, MDM2, hnRNPA2B1, and NICD), and further explored the potential application of combining TAAbs with miRNAs in detection of ESCC.

## 2 METHODS AND MATERIALS

### 2.1 Plasma samples

From April 2013 to December 2014, plasmas from 125 patients diagnosed with ESCC without other malignancies were collected from the Henan Cancer Hospital (Zhengzhou, China). Diagnoses of ESCC were carried out by histopathology and none of the patients received surgical treatments, radiotherapy, or chemotherapy. A total of 125 healthy subjects without any disease, selected from the cardiovascular disease investigation (Henan Province, China), were matched to the ESCC patients by age and sex. The whole blood samples of the ESCC patients were assessed in EDTA‐K2 anti‐coagulant tubes. Plasma was centrifuged within 2 hours of collection at 3000 rpm for 5 minutes and then stored at −80°C for further use. This study was approved by the ethics committee of Henan Cancer Hospital and Zhengzhou University and all participants signed informed consent.

### 2.2 Experimental methods

The seven TAAbs were detected in plasma by indirect sandwich enzyme immunoassay technique. We used 0.5 μg/mL purified recombinant protein to coat each well of a 96‐well ELISA plate and used human IgG protein (Beijing Dingguo Changsheng Biotechnology) as a standard reference. The IgG dry powder was diluted to eight standard concentrations of 10, 20, 50, 100, 150, 200, 250, and 300 ng/mL to obtain a standard curve for each plate. The detail steps of ELISA were described in our previous study.^24^ Based on the standard curve of each plate, the OD value of each autoantibody was converted to the expression concentration. The RNA purification and reverse transcription quantitative‐polymerase chain reaction (RT‐qPCR) have been shown in a previous study.^23^ The expression levels of five miRNAs were calculated with the equation of 2^–ΔCt^ (ΔCt ESCC = Ct _ESCC_ − Ct _5S rRNA_, ΔCt control = Ct _control_ − Ct_5S rRNA_).

### 2.3 Statistical analysis

IBM SPSS Statistics software (version 21.0) and GraphPad Prism 6.0 were used to analyze the data. The Mann‐Whitney *U* test (nonparametric tests) was used to analyze the differences of biomarkers between two groups, because the expression levels of these markers showed abnormal distribution (Kolmogorov‐Smirnov test). The ROC analysis was applied to estimate the diagnostic value of each marker for distinguishing ESCC from normal individuals, and the sensitivity is determined by specificity more than 90%. Logistic regression models were constructed under the forward condition to predictive the risk of ESCC based on expression levels of markers in ESCC patients and normal controls. The predicting probability *P* = .5 was defined as cutoff point to calculate the positive predictive value (PPR), negative predictive value (NPR), positive likelihood ratio (PLR) and negative likelihood ratio (NLR), and accuracy, and was used as a surrogating marker to protract ROC curve.^25^ All *P* values were calculated based on two‐tailed and *P* < .05 was determined to be significant.

## 3 RESULTS

### 3.1 Characteristics of ESCC patients and normal controls

A total of 125 patients with ESCC and 125 healthy controls were entered in this study. The characteristics of all patients and controls were described in Table 1. Statistical analysis showed that there was no significant difference in age and sex distribution in ESCC and normal controls (*P* > .05). The patients with ESCC were staged according to the TNM staging standard of the seventh edition of UICC.^26^ TNM staging was stages 0, I, II, III, and IV of the ESCC patients accounted for 5.6%, 37.6%, 19.2%, 30.4%, and 4.0%, respectively. Stage TNM 0‐I or Tis‐1 was defined as early stage ESCC according to UICC.

**Table 1:** Patient details and clinicopathological characteristics

	ESCC (n = 125)		Normal (n = 125)	
	N	%	N	%
Sex				
Male	76	60.8	76	60.8
Female	49	39.2	49	39.2
Age (y)				
Range	41‐80		40‐79	
Median (P_25_ ~ P_75_)	63 (59 ~ 68)		63 (59 ~ 68)	
Site of tumor				
Upper esophagus	22	17.6		
Middle esophagus	65	52		
Lower esophagus	31	24.8		
Unknown	7	5.6		
Histological grade				
High (grade 1)	15	12		
Middle (grade 2)	46	36.8		
Low (grade 3)	50	40		
Unknown	14	11.2		
TNM stage				
0	7	5.6		
I	47	37.6		
II	38	30.4		
III	24	19.2		
IV	5	4		
Unknown	4	3.2		
Depth of tumor invasion				
Tis	7	5.6		
T1	43	34.4		
T2	20	16		
T3	48	38.4		
T4	0	0		
Unknown	7	5.6		
Lymph node metastasis				
Negative	86	68.8		
Positive	32	25.6		
Unknown	7	5.6		
Distant metastasis				
No	116	92.8		
Yes	5	4		
Unknown	4	3.2		

Abbreviations: ESCC, esophageal squamous cell carcinoma; P_25_, upper quartile, P_75_, lower quartile.

### 3.2 Diagnostic value of TAAbs in patients with ESCC

**Figure 1:**
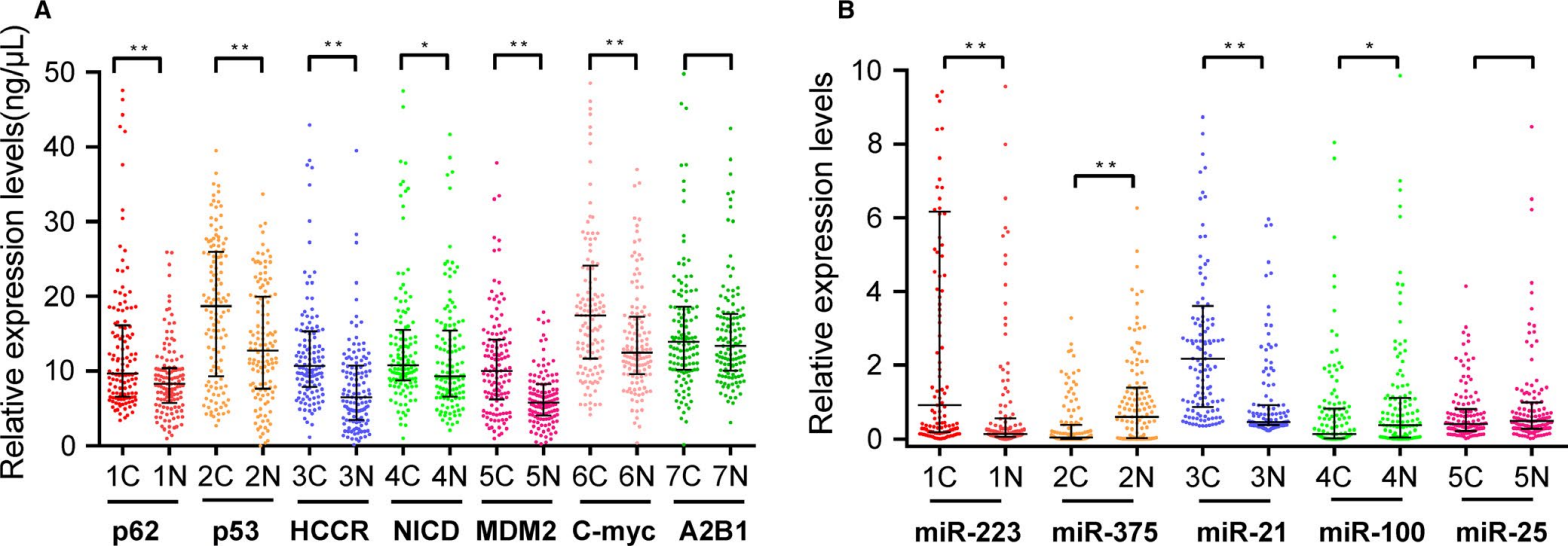
The expression levels of TAAbs and miRNAs in esophageal squamous cell carcinoma (ESCC) patients and healthy controls. a scatter plots of the expression levels of autoantibodies in 125 ESCC patients and 125 healthy controls, b scatter plots of the expression levels of miRNAs in 125 ESCC patients and 125 healthy controls. Line, median with interquartile range; C, cancer; H, healthy. *P < .05, **P < .01(Mann‐Whitney *U* test)

**Figure 2:**
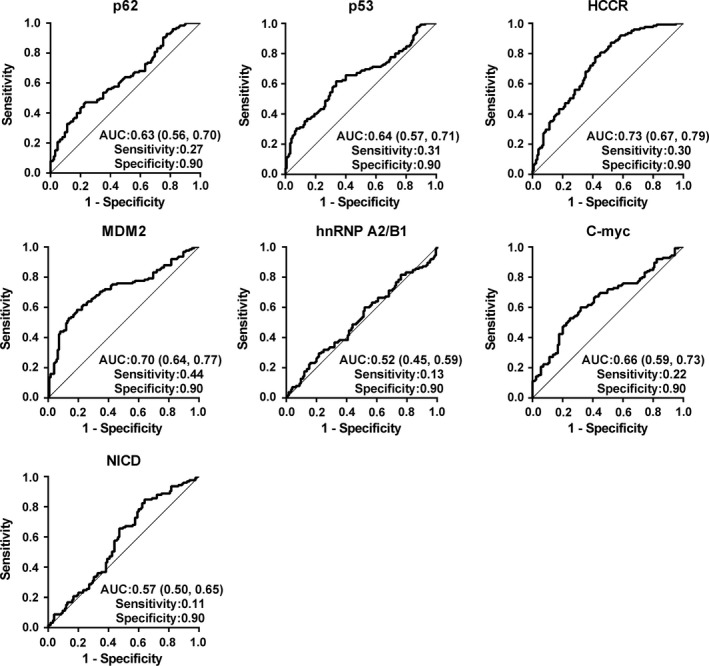
Performance of seven TAAbs for discriminating esophageal squamous cell carcinoma patients from healthy individuals

The expression levels of seven TAAbs in cases and normal individuals were illustrated in Figure 1A. The expression levels of p53, p62, HCCR, C‐myc, NICD, and MDM2 in ESCC patients were significantly higher than those in controls (*P* < .05). However, the median value of hnRNPA2B1 for ESCC patients was 13.89 ng/µL (range, 10.20 ng/µL to 18.63 ng/µL), and healthy controls was 13.34 ng/µL (range, 10.05 ng/µL to 17.49 ng/µL). Thus, these differences were not significant between the two groups (*P* = .547). To evaluate the diagnostic value of autoantibodies in patients with ESCC, we applied ROC curve to determine the sensitivity according to specificity more than 90%. The diagnostic performances of single TAAb were shown in Figure 2, and the AUCs ranged from 0.52 to 0.73. Although patients with ESCC could be distinguished from healthy individuals, single TAAb showed poor diagnostic ability. The AUC of anti‐HCCR autoantibody in diagnosis of ESCC was the largest among seven TAAbs and reached 0.73 (95% CI: 0.67‐0.79), but the sensitivity was just 30% when the specificity was 90%.

**Figure 3:**
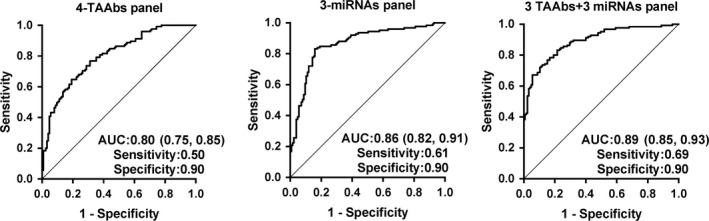
Performance of the different prediction model panels to detect esophageal squamous cell carcinoma. Four TAAbs including p53, HCCR, C‐myc, and MDM2; three miRNAs including miR‐21, miR‐100, and miR‐375; 3TAAbs + 3miRNAs including HCCR, C‐myc, MDM2, miR‐21, miR‐223, and miR‐375

**Figure 4:**
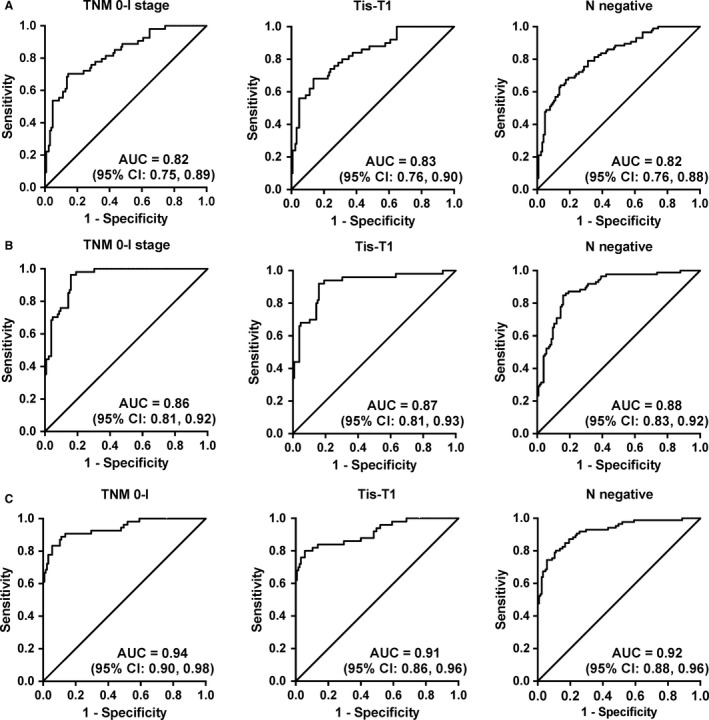
The diagnostic value of different panels for early esophageal squamous cell carcinoma patients. (A) The diagnostic value of TAAb panel (p53, HCCR, C‐myc, and MDM2), (B) The diagnostic value of miRNAs panel (p53, HCCR, C‐myc, and MDM2), (C) The diagnostic value of combining TAAb with miRNAs panel (HCCR, C‐myc, MDM2, miR‐21, miR‐223, and miR‐375)

Logistic regression analysis was employed to identify optimal panels for detecting patients with ESCC. Based on the expression levels of six TAAbs (p53, p62, HCCR, NICD, C‐myc, and MDM2) with statistical significance in two groups, the regression model consisting of four TAAbs (p53, HCCR, C‐myc, and MDM2) can classify the individuals correctly with an accuracy of 72%. The possibility for diagnosis as ESCC was PRE (P = ESCC, 4 TAAbs) = 1/(1 + EXP (−(−3.408 + 0.095 × HCCR+0.109 × MDM2 + 0.045 × C‐myc + 0.052 × p53))). The model had AUC of 0.80 (95% CI: 0.75‐0.86) with the sensitivity of 50% and specificity of 90% (Figure 3 and Table 2). Compared with single TAAbs, the AUC of four TAAbs was significantly higher than the AUC of any of seven TAAbs. The diagnostic value of the panel of four TAAbs for early ESCC was similar. The AUCs of TNM0‐I, Tis‐T1, and negative lymph node metastasis were 0.83, 0.83, and 0.82, respectively (Figure 4 and Table 2). Furthermore, the diagnostic performance of the combination for early ESCC was higher than that for all‐stage patients, which mainly reflected in AUC and sensitivity (Table 2).

**Table 2:** The diagnostic value of different models for detecting ESCC

Panels	Stage	AUC (95% CI)	Se (%)	Sp (%)	Accuracy (%)	PPV (%)	NPV (%)	PLR	NLR
Four TAAbs (p53, HCCR, C‐myc, and MDM2)	All stage	0.80 (0.75,0.85)	49.60	90.40	71.60	73.28	70.15	3.38	0.44
	TNM0‐I	0.83 (0.76,0.89)	55.56	90.40	74.30	55.71	40.17	4.89	0.35
	Tis‐T1	0.83 (0.77,0.90)	58.00	90.40	74.86	54.41	40.52	5.00	0.37
	N negative	0.82 (0.76,0.88)	53.49	90.40	73.46	66.30	38.52	3.83	0.40
Three miRNAs(miR‐21, miR‐100, and miR‐375)	All stage	0.86 (0.82,0.91)	60.80	90.40	77.20	85.42	72.08	5.20	0.20
	TNM0‐I	0.94 (0.91,0.97)	75.93	90.40	84.92	74.55	44.58	6.02	0.04
	Tis‐T1	0.91 (0.86,0.96)	68.00	90.40	83.43	71.43	44.22	5.75	0.10
	N negative	0.89 (0.85,0.94)	65.12	90.40	80.09	80.56	42.04	5.30	0.18
3TAAbs + 3miRNAs (HCCR, C‐myc, MDM2 miR‐21, miR‐223, and miR‐375)	All stage	0.89 (0.85,0.93)	68.80	90.40	80.80	83.48	78.52	3.85	0.21
	TNM0‐I	0.94 (0.90,0.98)	83.33	90.40	86.59	72.06	44.92	14.96	0.17
	Tis‐T1	0.91 (0.86,0.96)	80.00	90.40	84.57	68.85	44.35	14.29	0.21
	N negative	0.92 (0.88,0.96)	75.58	90.40	83.89	78.89	43.09	7.16	0.22

Abbreviations: AUC, area under the receiver operating characteristic curve; CI, confidence interval; miRNAs, microRNAs; N negative: negative lymph node metastasis; NLR, negative likelihood ratio; NPV, negative predictive value; PLV, positive likelihood value; PPV, positive predictive value; Se, sensitivity; Sp, specificity; TAAb, autoantibodies against tumor‐associated antigens.

### 3.3 Diagnostic performances of miRNAs in patients with ESCC

The expression levels of miRNA‐21, miRNA‐375, miRNA‐223, miRNA‐100, and miRNA‐25 in plasma were detected by qRT‐PCR in 125 ESCC patients and 125 matched controls. The levels of five miRNAs in the ESCC group and controls group were exhibited in Figure 1B. There was a significant difference in the expression levels of miR‐21, miR‐223, miR‐375, and miR‐100 between cancer cases and control individuals. The single diagnostic value of five miRNAs has been shown in the previous study, and the AUCs of single miRNAs ranged from 0.56 to 0.80 for ESCC patients.^23^

In this study, we mainly estimate the diagnostic value of combined miRNAs in ESCC. Based on the expression levels of four significant miRNAs, the model including three miRNAs (miR‐21, miR‐100, and miR‐375) was generated by logistic regression analysis, with the accuracy of 77% for diagnosing cancer patients. The possibility for diagnosis as ESCC was PRE (*P* = ESCC, 3 miRNAs) = 1/(1 + EXP (−(−0.693 + 0.793 × miR‐21 + 0.231 × miR‐100‐1.209 × miR‐375))). The panel had AUC of 0.86 (95% CI: 0.82‐0.91) with the sensitivity of 61% and specificity of 90% (Figure 3 and Table 2). The diagnostic sensitivity of the panel for cancer patients with TNM0‐I, Tis‐T1, and negative lymph node metastasis was 76%, 68%, and 65%, respectively (Figure 4 and Table 2).

### 3.4 Evaluation of the diagnostic value of combining TAAbs and miRNAs for ESCC detection

Compared with single autoantibodies or miRNAs, the combination of plasma miRNAs or TAAbs has higher diagnostic value for ESCC. Therefore, this study considered combining miRNAs and TAAbs to evaluate the combined diagnostic value of two types of tumor markers in ESCC. Based on the expression levels of these nine significant markers (p53, p62, HCCR, C‐myc, MDM2, miR‐21, miR‐223, miR‐375, and miR‐100), we found that a panel of six plasma markers (HCCR, C‐myc, MDM2, miR‐21, miR‐223, and miR‐375) was able to effectively discriminate the ESCC patients from control individuals. The predicted probability for detecting ESCC was as follows: PRE (*P* = ESCC,6‐makers) =1/(1 + EXP (−(−3.592 + 0.12 × HCCR+0.086 × MDM2+0.052 × C‐myc + 0.799 × miR‐21 + 0.127 × miR‐223 − 0.942 × miR‐375))). The ROC curve showed that the panel had AUC of 0.89 (95% CI: 0.85‐0.93) with the sensitivity of 69%, the specificity of 90%, the PPV of 83%, the NPV of 79%, the PLR of 3.9, the NLR of 0.2, and the coincidence rate of 81% (Figure 3 and Table 2).

The diagnostic accuracy of the six‐member panel of markers (HCCR, C‐myc, MDM2, miR‐21, miR‐223, and miR‐375) for cancer patients with TNM0‐I, Tis‐T1, and negative lymph node metastasis was 87%, 85%, and 84%, respectively (Figure 4 and Table 2). More importantly, this panel distinguished stage TNM0‐I and Tis‐T1 ESCC patients from controls. The panel of six markers provided an enhanced accuracy of 87%, sensitivity of 83%, specificity of 90%, and PLR of 15 in the diagnosis of TNM 0‐I ESCC (Figure 4). The panel exhibited an accuracy of 85% for patients with stage Tis‐T1, with the sensitivity of 80%, specificity of 90%, and PLR of 14 (Table 2).

## 4 DISCUSSION

In this study, we firstly explored the diagnostic value of combining TAAbs with miRNAs for ESCC patients. Three panels (four TAAbs, three miRNAs, and three TAAbs+ three miRNAs) were constructed based on the logistic regression analysis. The combination of multiple biomarkers could attain higher sensitivity compared with single biomarkers, which was consistent with the published data.^27^, ^28^, ^29^ Importantly, the further results indicated that the diagnostic value could be greatly improved via the combination of miRNAs and TAAbs, with AUC of 0.89 (0.85‐0.93), sensitivity of 69%, specificity of 90%, and the accuracy of 81%, which was hopeful to surmount the tricky problem of low sensitivity of single type molecular marker. Combined with the clinical stages of patients with ESCC, we found that the optimal model (p53, p62, HCCR, C‐myc, MDM2, miR‐21, miR‐223, miR‐375, and miR‐100) can provide higher diagnostic value for early ESCC, such as TNM0‐I, Tis‐T1, and no regional lymph node metastasis (N‐staging negative). Compared with a single type of combination of miRNAs or TAAbs, the combination of miRNAs and TAAbs can effectively improve the diagnostic value of ESCC.

The positive rates of single markers are too low to meet the need for clinical practice. In present study, six autoantibodies (NICD, p53, p62, HCCR, C‐myc, and MDM2) were identified from developed ESCC. The AUC of six anti‐TAA autoantibodies was 0.57‐0.73 with the sensitivity of 11%‐44% and the specificity of 90%. The diagnosis value of single TAAbs for cancer was limited, which was consistent with the results reported in the literature.^30^, ^31^ Since clinical practice is based on more than one diagnostic technique, higher sensitivity will indicate doctors to choose further diagnostic tools. It is worth learning that the detection system using antigen platform includes several TAAs, which can increase the sensitivity for detecting cancer.^28^ Comparatively, the panel of our four TAAbs (p53, HCCR, C‐myc, and MDM2) reached a higher sensitivity of 50% with specificity of 90%. Correspondingly, the consistent rate of a panel of three miRNAs (miR‐21, miR‐100, and miR‐375) reached 77%, which indicated that combined miRNAs for detection of ESCC had greater validity, especially for the patients with TNM 0‐I or Tis‐T1 stage.

Sensitivity and specificity largely depend on the cutoff point. Many researchers defined the mean plus two or three standard deviations of the controls as the cutoff value, which likely leads to rather a high specificity and insufficient sensitivity.^32^, ^33^ Of note, when sensitivity and specificity are calculated by ROC curves, the cutoff value is not considered,^34^ which brings great convenience for comparing the diagnostic ability of between different markers and achieves the purpose of screening more accurately.^35^ In this study, we used ROC curves to assess the diagnostic performance of established models. The three models—three miRNAs, four TAAbs, and three miRNAs combined with three TAAbs—showed AUC of 0.86 (95% CI: 0.82‐0.91), 0.80 (95% CI: 0.75‐0.86), 0.89 (95% CI: 0.85‐0.93), separately. The results showed that the diagnostic value of the combined detection of two types of markers was improved in sensitivity (from 50% to 68%), AUC (from 0.80 to 0.89), and coincidence rate (from 72% to 81%) to a certain extent compared with the combination of markers of single type.

Our study presented that the six‐member panel may be used as a tool for diagnosing ESCC and importantly has the potential to predict ESCC patients at a relatively early stage. The specificity and sensitivity of this optimal panel were more than 90% and 65%, respectively. The likelihood ratio was an index reflecting validity, which comprehensively reflected the sensitivity and specificity. The positive likelihood ratio was the ratio of true positive rate to a false positive rate of screening results. The greater the ratio, the greater the probability that a positive result in the screening test will be a true positive. The positive likelihood ratio of this panel was significantly higher than that of single type of marker combination.

We firstly reported the diagnostic value of combining miRNAs and TAAbs in esophageal cancer and achieved promising results. But there are also some limitations in our research. First, the sample size was not large enough, and only 125 pairs of age‐and gender‐matched subjects were recruited. Second, we did not combine traditional cancer markers with selected markers in this study, such as CEA, SCC‐Ag, or CYFRA21‐1. Dong et al demonstrated that a significantly higher number of ESCC patients were detected positive for combing CDC25B‐Abs, CEA, SCC, and CYFRA21‐1 (64.2%) compared to the panel of CEA, SCC‐Ag, and CYFRA21‐1 (41.0%).^36^

In summary, our results reveal that using the combination of miRNAs and TAAbs may aid to diagnose ESCC, especially for ESCC with the early stage. In addition, our research results also provide new ideas for researchers engaged in cancer diagnosis. Without doubt, more investigation and the testing of large‐scale ESCC samples are needed to prove the result for possible application in clinical practice.

## CONFLICT OF INTEREST

All authors declare no conflict of interest.

## Data Availability

The data that support the findings of this study are available from the corresponding author upon reasonable request.
